# Cancer of the breast: 5-year survival in a tertiary hospital in Uganda

**DOI:** 10.1038/sj.bjc.6604435

**Published:** 2008-06-24

**Authors:** A Gakwaya, J B Kigula-Mugambe, A Kavuma, A Luwaga, J Fualal, J Jombwe, M Galukande, D Kanyike

**Affiliations:** 1 Surgery Department, Mulago Hospital, PO Box 7051, Kampala, Uganda; 2 Radiotherapy Department, Mulago Hospital, PO Box 7051, Kampala, Uganda

**Keywords:** carcinoma breast, radiotherapy, surgery, chemotherapy, 5-year survival

## Abstract

The objective was to investigate survival of breast cancer patients at Mulago Hospital. A retrospective study of the medical records of 297 breast cancer patients referred to the combined breast clinic housed in the radiotherapy department between 1996 and 2000 was done. The female/male ratio was 24 : 1. The age range was 22–85 years, with a median of 45 years and peak age group of 30–39 years. Twenty-three percent had early disease (stages 0–IIb) and 26% had metastatic disease. Poorly differentiated was the most common pathological grade (58%) followed by moderately differentiated (33%) and well-differentiated (9%) tumours. The commonest pathological type encountered was ‘not otherwise specified’ (76%). Of all patients, 75% had surgery, 76% had radiotherapy, 60% had hormonotherapy and 29% had chemotherapy. Thirty-six (12%) patients received all the four treatment modalities. The 5-year survival probabilities (Kaplan–Meier) for early disease were 74 and 39% for advanced disease (*P*=0.001). The overall 5-year survival was 56%, which is lower than the rates in the South African blacks (64%) and North American whites (82–88%).

Cancer of the breast is a major health burden and the most common cancer in women worldwide. It is among the most common causes of cancer death in women in both high-resource and low-resource settings, and is responsible for over 1 million of the estimated 10 million neoplasms diagnosed worldwide each year in both sexes (Ferlay *et al*, 2001). The incidence in developed countries, for example, the United States and the United Kingdom, is much higher, ranging between 50 and 100 per 100 000 (Althuis *et al*, 2005), and was responsible for about 375 000 deaths in the United States in 2000 (Bray *et al*, 2004).

Breast cancer is the third most common cancer in women in Uganda after cancer of the cervix and Kaposi's sarcoma (Parkin *et al*, 1997). Breast cancer incidence in Uganda was 11 : 100 000 in 1961 and had doubled to 22 : 100 000 by 1995 (Wabinga *et al*, 2000). The incidence in Zimbabwe is 20, in Gambia 4, in South Africa 70 (in whites) and 11 (in blacks) per 100 000 (Vorobiof *et al*, 2001).

African women are diagnosed more often between 35 and 45 years, which is more than 15 years earlier than the women in Europe and North America. The mortality rate in sub-Saharan Africa is disproportionately high compared with the incident rate (Fregene and Newman, 2005).

Five-year overall survival rate varies from 86% in Canada (NCIC, 2006) to 88% in the United States (ACS, 2006), whereas it was 80% in whites and 64% in blacks in South Africa (Vorobiof *et al*, 2001).

The main objective of this study was to investigate the 5-year overall survival of breast cancer patients treated at Mulago Hospital, which is a national referral and tertiary centre.

## Patients and methods

This was a retrospective study of 297 patients with histologically diagnosed cancer of the breast referred to the combined multidisciplinary breast clinic, which is housed in the radiotherapy department, from 1996 to 2000. An attempt to retrieve files of other patients who might not have been referred to the combined breast clinic was made but none was found. Patients' files were manually retrieved. All patients' records, irrespective of their co-morbidities, performance status, stage and treatment modalities, were included. A review of their medical records was done in July 2006. The following data were collected from their medical records:

sex;
age of the patients at presentation;
stage at diagnosis (AJCC, 2002);
histological types;
histological grades;
treatment modality given to the patient, for example, surgery, radiotherapy, chemotherapy and hormonotherapy. Those who did not receive any of these were also noted;
time in months when they died or got lost or if they were still on follow-up.

### Statistical analysis

The survival probabilities were worked out using the Kaplan–Meier method of estimating the survival. All patients who received any of the following modalities of treatment (surgery, chemotherapy, radiotherapy and hormonotherapy) were analysed when estimating survival. Survival rate was taken as the proportion of people diagnosed with a disease who live for a specific period of time (ACS, 2006).

The 5-year cancer survival rate was therefore taken as the proportion of cancer patients who were still alive, not necessarily disease-free, 5 years after the diagnosis of cancer was made.

Twelve patients (4%) did not receive any of the above treatments. They were not included in the calculation of the survival rates.

A *P*-value of <0.05 was considered to denote statistical significance. Histograms were used to present continuous variables and tables used for categorical data.

## Treatment policy

Patients were referred by surgeons to a combined multidisciplinary clinic, which has been meeting every Friday for the past 13 years in the radiotherapy department. Final decisions on adjuvant treatment were made during this meeting. We followed our treatment guidelines, which were later published by The Uganda Cancer Working Group (2003).

Surgical treatment was breast-conserving surgery, mastectomy and auxiliary clearance for early disease. Toilet mastectomy was performed in late disease stage when indicated. Combination chemotherapeutic agents using cyclophosphamide, adriamycin and 5-fluorouracil (CAF) or cyclophosphamide, methotrexate and 5-fluorouracil (CMF) were used. Radiotherapy was either radical (50–66 Gy) or palliative (8–30 Gy) using a cobalt-60 teletherapy beam. A dose of 50 Gy in 25 fractions over a 5-week period was used after mastectomy to the chest walls using two tangential fields. A similar dose was delivered to the supraclavicular and auxiliary lymph node regions when indicated. In cases of breast-conserving surgery, a booster dose of 16 Gy in eight fractions was given to the tumour bed. In palliative situations, a dose range of 8 Gy in single fraction to 30 Gy in 10 fractions was given to either the breast or the metastatic lesion(s). Hormonotherapy (tamoxifen) was offered to all patients with a daily dose of 20 mg for at least 5 years as oestrogen receptors (ER), and progesterone receptors (PR) were not tested. Patients had to buy the chemotherapy drugs and tamoxifen, as they were not available in the hospital.

## Results

A total of 297 patients' files were retrieved and all were analysed.

### Basic demographics

#### Age

**Figure 1:**
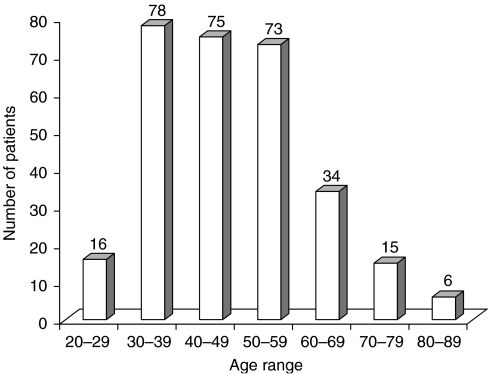
Age distributions.

The peak age group was 30–39 years. The median age was 45 years, mode 50 years and mean 47 years with a range of 22–85 years. Figure 1 shows the age distribution of the patients.

#### Sex

There were 285 (96%) female patients and 12 (4%) male patients. The male/female ratio was 1 : 24.

#### Stage

**Figure 2:**
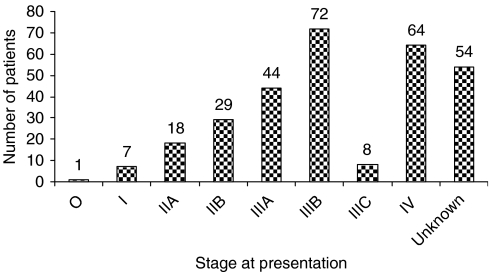
Stage at presentations.

The stage was established in 243 (82%) patients. Stage III was the peak stage at presentation with 51% of all patients; 26.3% of the patients were metastatic at presentation. Only 23% were early cancers (stages 0–IIb). Figure 2 shows the stage of the patients at presentation.

#### Histological types

Histological types were available for 122 out of 297 (41%) patients. The most common histological type was ‘infiltrating ductal carcinoma – not otherwise specified’ making up 76% of the patients. Table 1 shows the histological types of patients.

#### Histological grades

**Figure 3:**
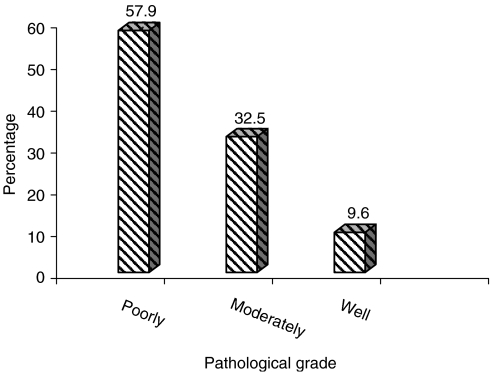
Pathological grades.

Histological grades were available for 83 out of 297 (28%) patients. Of these, 58% were poorly differentiated. The histological grades are shown in Figure 3.

#### Treatment

Two hundred and twenty-two (75%) patients received surgery (mastectomy and auxiliary clearance, toilet mastectomy and breast conservative surgery).

A total of 227 (76%) patients received external radiotherapy with a cobalt-60 beam. All the 15 patients (5%) who underwent breast-conserving surgery received a total dose of 66 Gy to the tumour bed with good cosmetic results. We did not have any incidence of salvage surgery after this treatment. Palliative radiotherapy was given to the 26% who had metastatic disease. This palliative treatment resulted in good response regarding pain relief and tumour regression in 80% of the patients treated. Spinal cord compression was the most common problem in the patients who presented with metastatic disease.

Seventy patients did not receive any of the radiation therapy. In 54 patients, the staging documentation was unclear from the records (Figure 2). These patients were part of the 70 patients who did not receive radiotherapy.

### Chemotherapy

**Figure 4:**
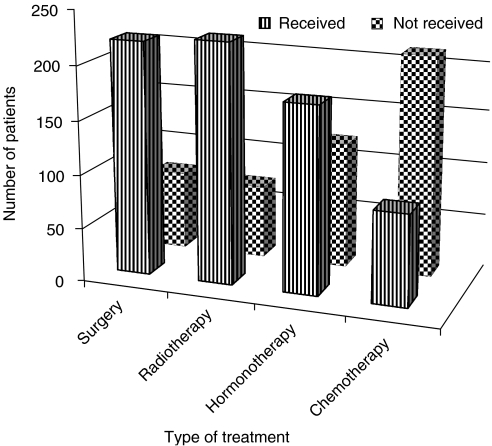
Treatment modalities.

Eighty-seven patients (29%) completed the six cycles of (CAF) chemotherapy as reflected in Figure 4. Thirty-one patients discontinued therapy after one or two cycles and these patients were not included in the histogram showing treatment modalities in Figure 4. They were, however, included in the calculations for the survival curves, as they received other treatments such as surgery and radiotherapy. One hundred and twenty patients never received chemotherapy, although they needed it according to our management guidelines. They, however, received other treatment modalities.

Figure 4 shows those who received the different treatment modalities.

In summary, 285 had some form of treatment; of these, 227 had radiotherapy, 222 had surgery, 177 had tamoxifen and 87 had chemotherapy.

Eighty-five patients died. Of the 285 patients who were eligible for analysis, only 79 (28%) were still being followed up at the end of the 5-year period.

### Five-year overall survival

**Figure 5:**
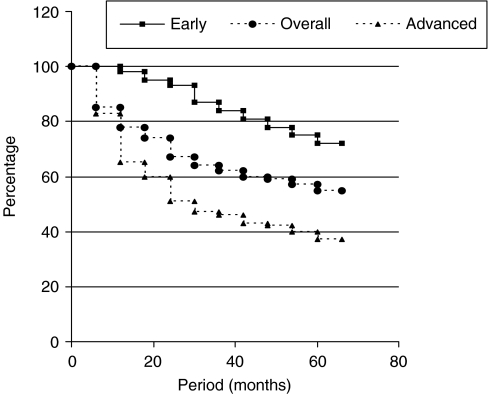
Survival probabilities (Kaplan–Meier).

The 2-year survival probabilities (Kaplan–Meier) were 94% for early disease stage and 56% for advanced disease stage (*P*=0.002), whereas those for 5-year survival were 74% for early disease stage and 39% for advanced disease stage (*P*=0.001). The overall 5-year survival was 56%. Figure 5 shows the percentage survival rates for early disease, advanced disease and for all patients who received some form of treatment.

## Discussion

### Five-year overall survival

Our 5-year survival rate was 74% for early disease stage and 39% for advanced disease stage. This is low compared with the American Cancer Society (2006) data , which were 97% for localised disease and over 47% for the late disease stage. Our 5-year overall survival rate, which was 56%, also compares unfavourably with those of the United States and Canada, which are 88 and 86%, respectively (ACS, 2006; NCIC, 2006). It is also low compared with the South African whites whose 5-year survival was 80%, but it is comparable with the survival in blacks, which was only 64% (Vorobiof *et al*, 2001).

These low survival rates may not be surprising considering that patients are disadvantaged regarding almost all the known prognostic factors, such as stage at diagnosis, histological grade and aggressiveness of the disease, availability and accessibility to appropriate treatment modalities and probably age at diagnosis.

### Stage at presentation

Bray *et al* (2004), while emphasising that prognosis is heavily dependent on stage of disease at presentation, points out that in the Surveillance, Epidemiology, and End Results (SEER) registries in the United States, 5-year survival for localised cases in 1994 was about 97% but was only about 25% for cases with metastatic disease.

Twenty-six percent of patients presented with metastatic disease. Even in the high-resource settings, treatment outcome is poor in metastatic disease. When metastatic disease is combined with stage III disease, they contributed more than 77% of all patients. This is late presentation compared with only 26% in Beirut (Saghir *et al*, 2006). In the United States, more than 22% of the patients present with carcinoma *in situ* disease. This is related to the rise in mammography-detected incidental cases as a result of the intensification of breast screening (Bray *et al*, 2004; ACS, 2006). There is, however, no screening programme in Uganda.

When one compares our survival rates with data from Nigeria, South Africa and Zimbabwe, it is noted that black Africans have three features in common: late stage in seeking treatment, lower age at peak incidence and severe tumour burden that takes a more aggressive course and consequently lower survival rates (Ihekwaba, 1992; Clayton and Byrd, 1993; Muguti, 1993; Moormeir, 1996; Ijaduola and Smith, 1998). In all these series, stages III–IV disease made up between 75 and 85% of all patients seen. It is, therefore, not surprising that Muguti (1993) reports a median survival of only 12 weeks in the 25 patients who were followed up to death.

We think that there are many socioeconomic factors that contribute to the late presentation of patients in Uganda. They include a low doctor/population ratio of 1 : 18750, a per capita income that is only USD 400 per annum, poor infrastructure, a low per capita health expenditure of $12 per annum, low literacy rate of 49% among females, stigma and inappropriate health-seeking behaviour where a person only goes to a health facility when he/she is critically sick.

### Histological grade and more aggressive disease

Fifty-seven percent of these tumours were poorly differentiated. This shows that a large proportion of patients had a more aggressive type of disease. This grade, with other parameters (tumour size and lymph node stage) remaining the same, has poor prognosis predictors/factors (D'Eredita *et al*, 2001). Although it is known that large tumours tend to be high grade and most tumours were of stage III, this percentage of poorly differentiated tumours must have contributed to the low 5-year survival rates. We were not able to compare this high proportion of poorly differentiated tumours with other series from Africa, as we did not find any report on pathologic grades.

A racial disparity in the progression of breast cancer has already been observed. This points to possible differences in the aetiology or in the genetic make-up of the two faces (Ochieng *et al*, 1997). This more aggressive nature of breast cancer in blacks can be further attributed to the fact that most tumours diagnosed are ER-negative (Chen *et al*, 1994; Gapsfar *et al*, 1996; Thomson *et al*, 2001). Oestrogen receptor-negative tumours tend to be more poorly differentiated and may have a shorter doubling time *in vivo* (Garret *et al*, 1993; Elias *et al*, 1994; Elledge *et al*, 1994). This may be an additional factor explaining why patients present with more advanced disease. We were not able to test for hormonal receptor status owing to limited resources and cannot confirm this correlation.

Of the total tumours, 76% was ‘infiltrating ductal carcinoma, not otherwise specified’ (IDC, NOS). This agrees with other literature (Chao *et al*, 1999), but it is higher than the 49% reported by Ihekwaba (1992) from Nigeria.

### Treatment

In high-resource to medium-resource settings, advances in breast cancer therapy in recent years have made a considerable contribution to improved survival and the subsequent reduction or stabilisation of breast cancer death rates. A most likely contributory factor to the decline, as noted in the United Kingdom, has been the establishment of treatment protocols, improved chemotherapeutic options or better therapeutic guidelines. Specific structural changes that have embraced specialisation of breast cancer care (such as, centralised treatment, adjustments in clinician workload and use of multidisciplinary teams) have been shown to improve outcome (Bray *et al*, 2004).

A combined multidisciplinary team has been meeting every week in Mulago hospital for the past 13 years and has been responsible for the production of the breast cancer guidelines for Uganda (The Uganda Cancer Working Group, 2003). We endeavoured to treat all the patients according to these guidelines despite resource limitations and difficulties with patient follow-up.

Surgery and radiotherapy were done according to our guidelines and these modalities were probably optimised. Of total patients, 75% underwent surgery. Of these, 58% had mastectomy plus auxiliary clearance (modified radical mastectomy), 35% had toilet mastectomy and 7% had conservative breast surgery. The percentage of patients who received breast-conserving surgery could have been higher, because up to 23% had stages 0–II disease. The knowledge that breast conservation is possible may have a multiplier effect on early presentations, as it encourages patients to come even earlier.

Fregene and Newman (2005) stated that treatment of breast cancer in sub-Saharan Africa is largely limited to surgery because of limited access to adjuvant therapy. Mastectomy is, therefore, the treatment of choice for the majority of cases. Another study (Muguti, 1993) cited the expense of travelling long distances to a hospital as the primary reason for patients having to restrict their therapeutic options. Both of these explanations may have contributed to the low percentage (29%) of patients who received chemotherapy.

Despite this, 76% of all patients received radiotherapy. This was radical for stages 0–III disease with an attempt to improve their survival, but only palliative treatment was given to the 26% who had metastatic disease.

Only 29% had chemotherapy (CAF). Even this traditional chemotherapy is too expensive for our population. A course of treatment costs about USD 250.

Although all the patients were given a prescription for tamoxifen, only 60% of them got this potentially useful treatment. It was not available in the hospital and the patients had to buy the medicine. Some could not afford this drug, which in developed countries is taken as a cheap drug and costs only about USD 4 per month in the open market.

A formula should, therefore, be found to improve access to these treatments. The new chemotherapies are not available.

### Age

The peak age in our series was 30–39 years, which is a young age group compared with 60–69 years in the United States (Pathak *et al*, 2000). The mean age at presentation of 47 years is comparable with the work done by Saghir *et al* (2006) in Beirut and Fregene and Newman (2005) in the United States. Young age at diagnosis of breast cancer has been reported in many studies from the United States and Europe to be an independent predictor of worse survival (Saghir *et al*, 2006). Breast cancers in Uganda occur in a younger population than those in the Western countries (Pathak *et al*, 2000) but are similar to the Nigerian and Zimbabwean situations (Ihekwaba, 1992; Muguti, 1993; Ikpatt *et al*, 2002). Our results show that 54% of all patients were below 50 years of age, whereas Swanson and Lin (1994) reported age incidence in the United States of less than 23% in the same age bracket. This means that when Uganda is planning strategies for detection, diagnosis, treatment and research, it should address the young. Ochieng *et al* (1997) and Garrett *et al* (1993)recommended that a cause for the age disparity should be looked for. They postulated a possible genetic or environmental cause. It is, however, possible that our data may not reflect a true increased incidence in the young women, as life expectancy is low in Uganda and only 4.2% of all women are above 50 years (UBOS, 2005). Despite this, we still think that our peak age of 30–39 years may be the youngest reported in literature.

### Sex

Of all cancer patients, 4% were male patients, which compares with Tanzania's 6.7% (Amir *et al*, 1996). These figures are very high compared with figures in Europe and the United States, where rates of less than 1% are quoted (Jonasson *et al*, 1996; Hill *et al*, 2005; ACS, 2006; NCIC, 2006). What causes the high rates of male breast cancer in Africa is still unknown. The absolute numbers of male breast cancers were too low for us to ascertain whether the behaviour of these tumours differed from that occurring in the females.

### Patient follow-up

This is difficult in our setting and may also contribute to the low survival rates, as some patients who may be taken as dead may still be alive. Although the Kaplan–Meier method makes allowance for these patients, still 46% of all patients lost in a 5-year period constitute a large percentage. The socioeconomic factors mentioned above compound the problem.

## Conclusions and recommendations

In conclusion, our study shows that the peak age was 30–39 years, 26% of the patients presented with metastatic disease, 58% were aggressive poorly differentiated tumours and that the overall 5-year survival rate was only 56%.

We recommend that a more detailed study to characterise breast cancer in the young African woman and a ‘National Cancer Control Programme’ be initiated to specifically improve access to diagnostic and therapeutic facilities in Uganda.

## Figures and Tables

**Table 1:** Histological types

**Type**	**Percent**
IDC (not otherwise specified)	76
Lobular	12
Schirrous	7
Others	5
